# Supplementary material for: Structure and Antiparasitic Activity Relationship of Alkylphosphocholine Analogues against Leishmania donovani
Source: Microorganisms. 2020 Jul 24;8(8):1117. doi: 10.3390/microorganisms8081117 (PMC7463460; doi:10.3390/microorganisms8081117)

Supplementary Data for: Structure and antiparasitic activity relationship of alkylphosphocholine analogues against *Leishmania donovani*.

Pages

2: Figure S1. Dose response curves of the activity of APC analogues against V82

3: Figure S2. Dose response curves of the activity of APC analogues Sb-resistant parasites

4: Figure S3. Dose response curves of the activity of APC analogues Milt-resistant parasites

5: Figure S4. Dose response curves and interaction profiles of mixed APCs against V82 promastigotes

6: Figure S5. Dose response curves and interaction profiles of mixed APCs against V82 intracellular amastigotes

Figure S1. Dose response curves of the activity of APC analogues. The activities of APC12, APC14, APC16, APC11UPC, APC11PC modified at the tail (a,d); APC126PC with linker comprised of 6 alkyl carbon (b,e); APC12 without the P-atom with one 12 alkyl carbon chain, DA, one 12 alkyl carbon chain, DAB or without the N-atom, PO (c,f) against promastigotes (a,b,c) 2or intracellular amastigotes (d,e,f) of *L. donovani* strain V82 respectively. Parasites were cultured in the presence of medium alone (controls, n=6) or compounds (n=3) at a concentration of 0.01-6.25µg/ml (promastigotes) and 0.01-0.197µg/l (intracellular amastigotes) for 72 hours post-treatment. The effect of drug treatment on parasite survival was determined by determining the mean suppression for experimental values compared to mean values. Data represents mean ± SE of three experiments.

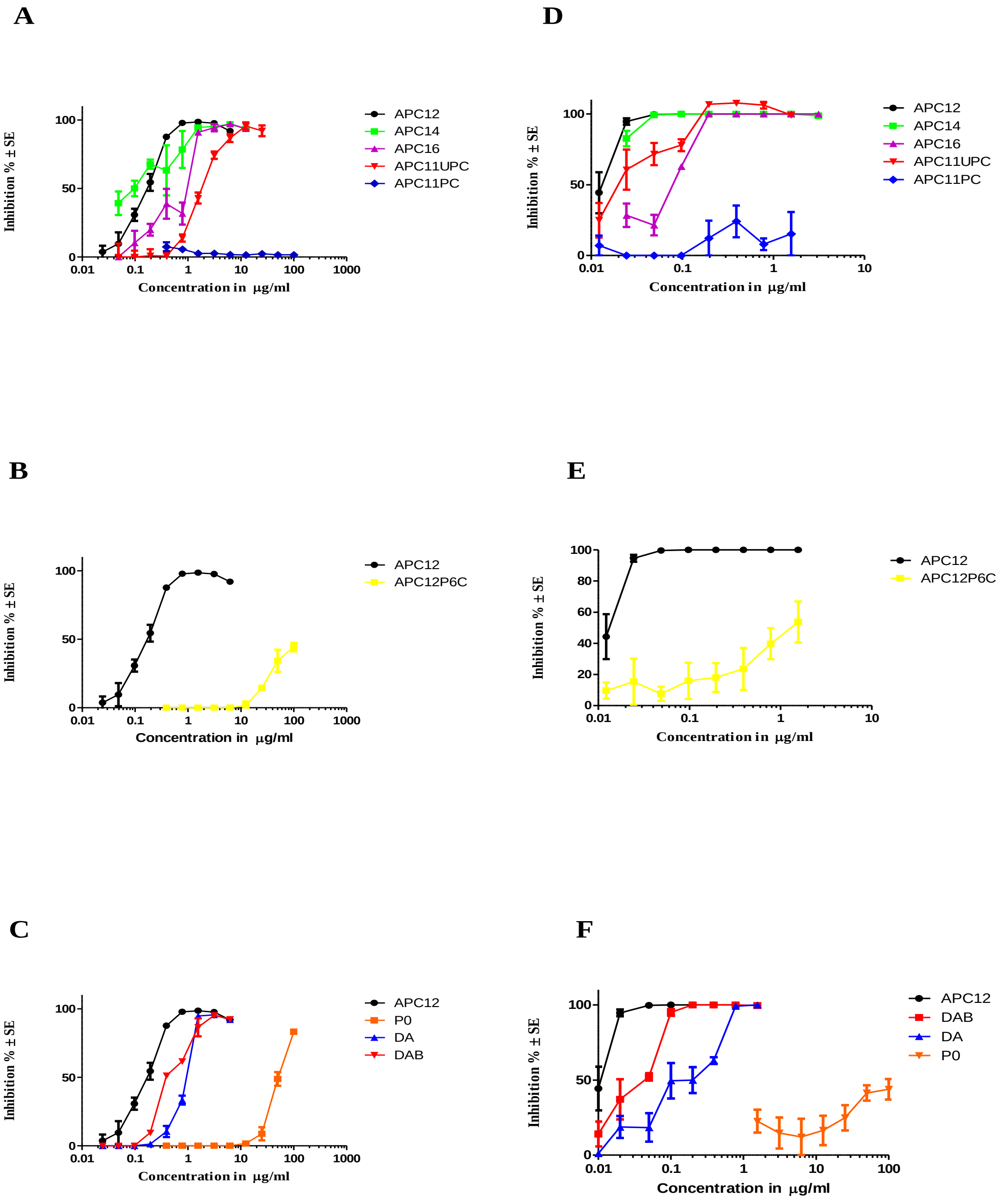

Figure S2. Dose response curves of the activity of APC analogues. The antileishmanial activity of APC12, APC14, APC16, APC18 against promastigotes (a,b,c) and intracellular amastigotes (d,e,f) against *L. donovani* strains with different antimony sensitivities (a, d, Sb sensitive; b, e Sb intermediate, c, f, Sb resistant). Parasites were cultured in the presence of medium alone (controls, n =6) or compounds (n=3) at a concentration of 0.01-6.25 µg/ml (promastigotes) or 0.01-0.197µg/ml intracellular amastigotes) for 72 hours post-treatment. The effect of drug treatment on parasite survival was determined by determining the mean suppression for experimental values compared to mean values. Data represents mean ± SE of three experiments. p<0.01(**) or p<0.005(***) of combined drugs relative to their individual counterpart.

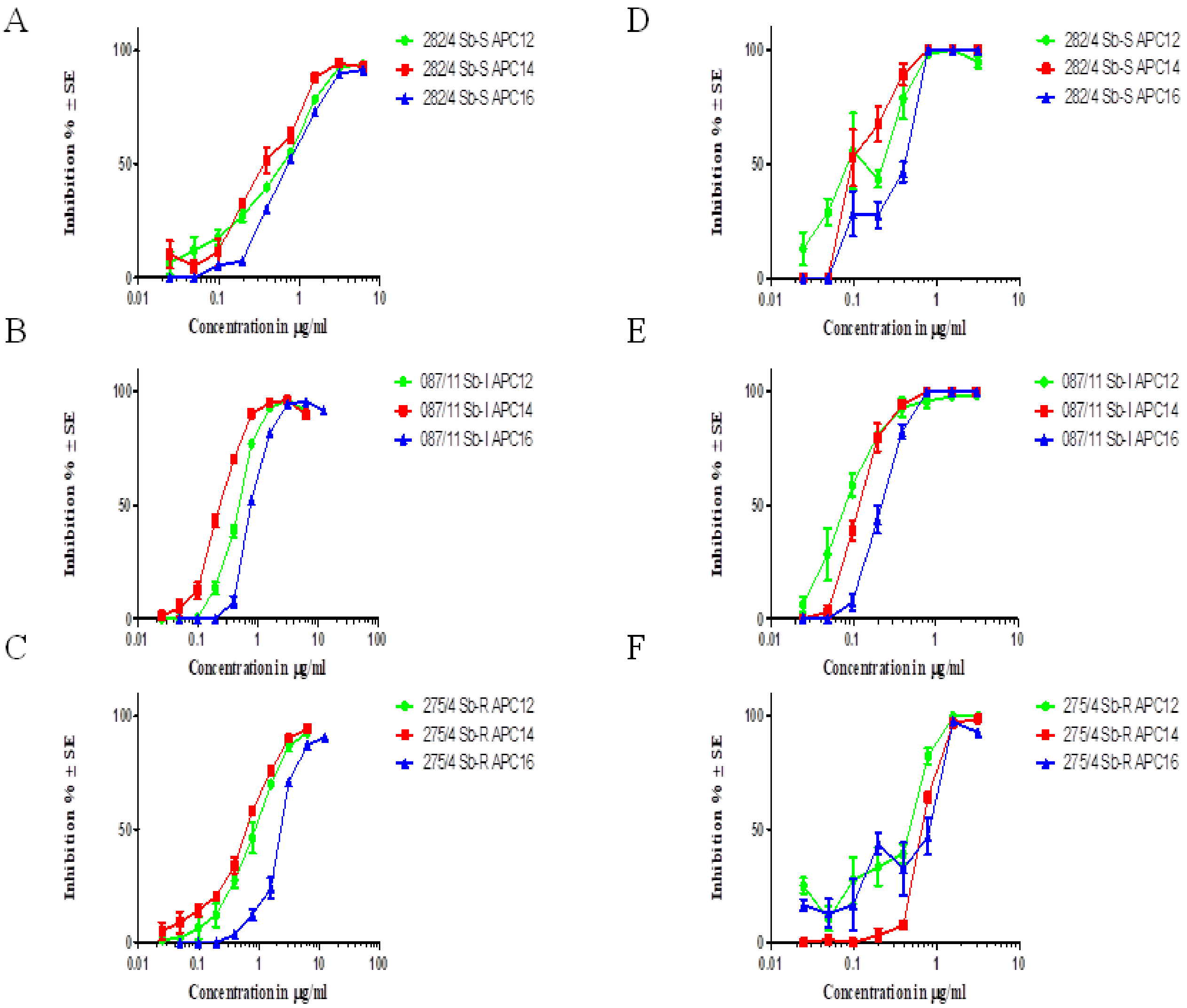

Figure S3. Dose response curves of the activity of APC analogues. The antileishmanial activity of APC12, APC14, APC16, APC18 against promastigotes (a,b,c) and intracellular amastigotes (d,e,f) against *L. donovani* Milt resistant strains against different antimony sensitivities (a, d, Sb sensitive; b, e Sb intermediate, c, f, Sb resistant). Parasites were cultured in the presence of medium alone (controls, n =6) or compounds (n=3) at a concentration of 0.01-6.25 µg/ml (promastigotes) or 0.01-0.197µg/ml intracellular amastigotes) for 72 hours post-treatment. The effect of drug treatment on parasite survival was determined by determining the mean suppression for experimental values compared to mean values. Data represents mean ± SE of three experiments. p<0.01(**) or p<0.005(***) of combined drugs relative to their individual counterpart.

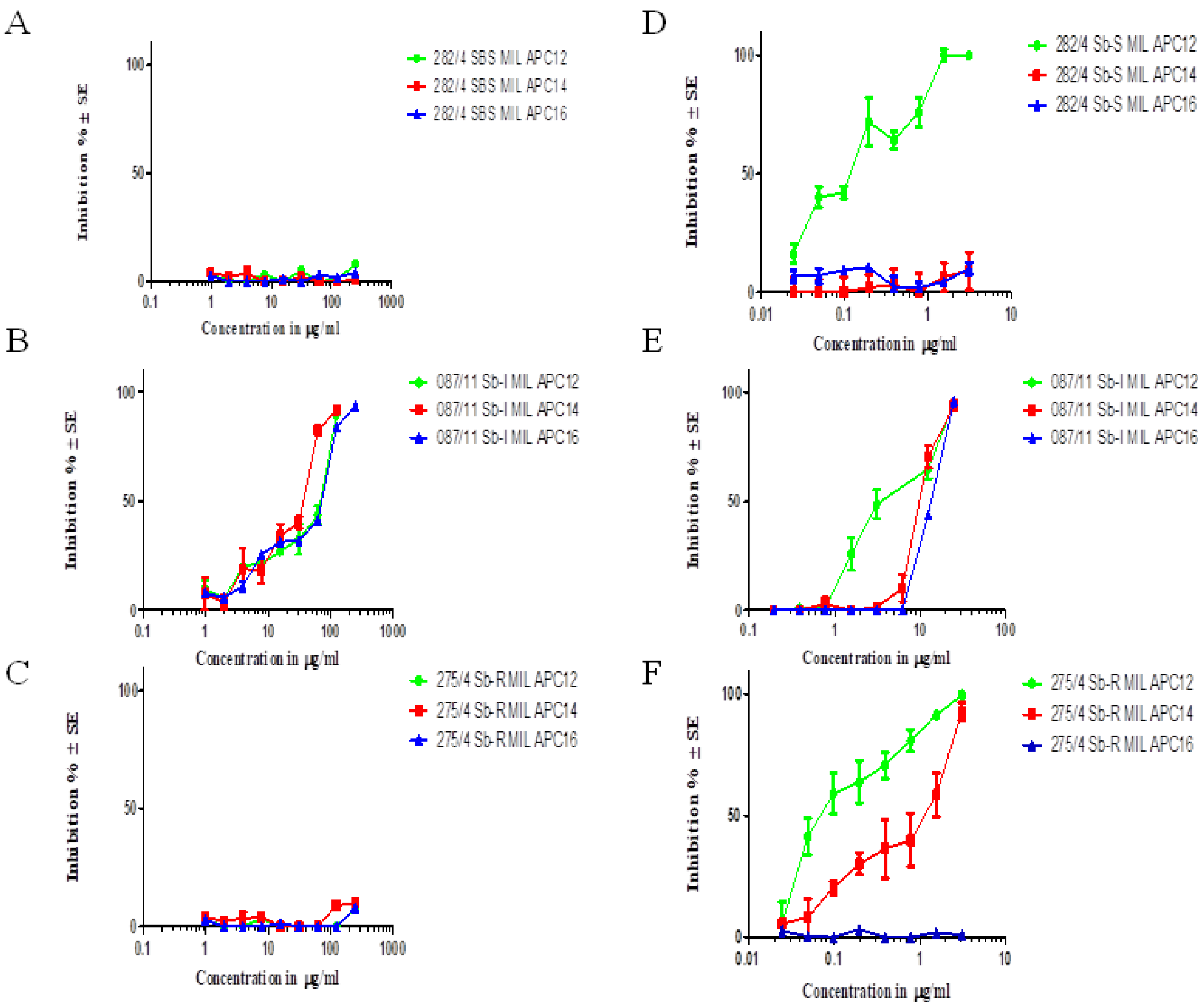

Figure S4. Dose response curves and interaction profiles of mixed APCs. The dose response curves (a,d), surface analysis (b,e) and heat map matrix (c,f) of varied concentrations of APC12 (0.01-6.25 µg/ml) with APC14 or APC16 at 0.195-0.39 µg/ml against *L. donovani* strain LV82 promastigotes (1×10^{7}) treated for 72 hours after drug treatment. The effect of drug treatment on parasite survival was determined by determining the mean suppression for experimental values compared to mean control value. Blue, green and red denotes synergistic, additive (green) and antagonistic interactions. The magnitude of the interaction is detailed in the heatmap. Compounds were tested using an n=3 and data are mean and standard deviations expressed as a percent of the control (parasites incubated with medium alone, n=6). Asterisks indicate significance at p<0.05(*), p<0.01(**) or p<0.005(***) of combined drugs relative to their individual counterpart.

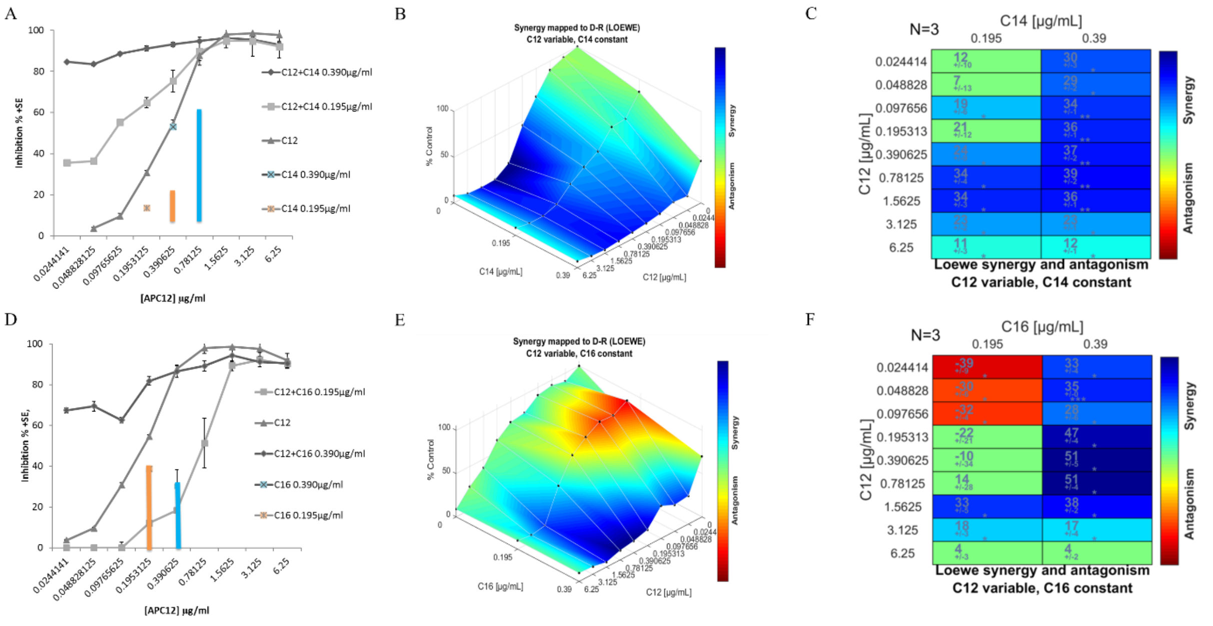

Figure S5. Dose response curves and interaction profiles of mixed APCs. The dose response curves (a,d), surface analysis (b,e) and heat map matrix (c,f) of varied concentrations of APC12 (0.01µg/ml-0.197µg/ml) interaction with APC16 or APC14 at 0.02µg/ml or 0.05µg/ml against *L. donovani* LV82 strain intracellular amastigotes treated for 72 hours after drug treatment. Viability were estimated using luciferase assay14. Blue, green and red denotes synergistic, additive (green) and antagonistic interactions. The magnitude of the interaction are detailed in the heatmap. Compounds were tested using an n=3 and data are mean and standard deviations expressed as a percent of the control (parasites incubated with medium alone, n=6). Asterisks indicate significance at p<0.05(*), p<0.01(**) or p<0.005(***) of combined drugs relative to their individual counterpart.

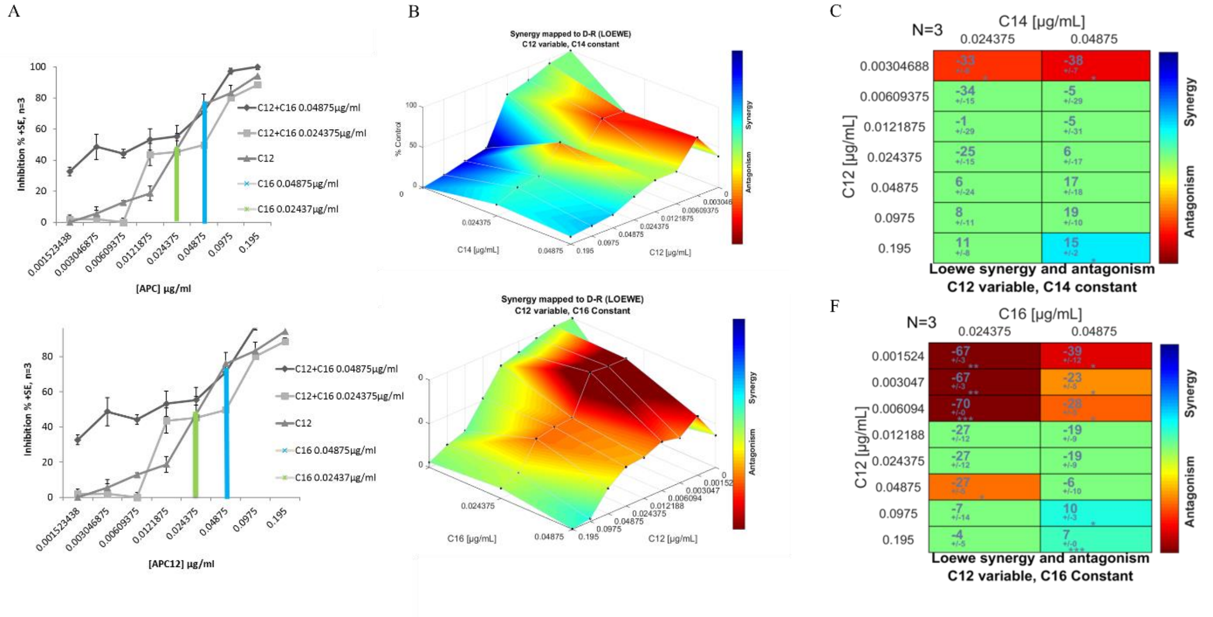

Supplement: Supplementary file 1 [file microorganisms-08-01117-s001.pdf]
